# COVID-19 Aortitis: A Review of Published Cases

**DOI:** 10.7759/cureus.22226

**Published:** 2022-02-15

**Authors:** Falah Abu Hassan, Minas Abu Alhalawa, Yacoub Majdoubeh, Ashish Nepal, Sareen S Sufan

**Affiliations:** 1 Internal Medicine, Texas Tech University Health Sciences Center, Amarillo, USA; 2 Neuroscience, Queen Mary University of London, Barts and The London School of Medicine and Dentistry, London, GBR; 3 Emergency Department, Kingston Hospital NHS Foundation Trust, London, GBR; 4 Internal Medicine, University Hospitals Sussex NHS Foundation Trust, Sussex, GBR; 5 Internal Medicine, The University of Jordan, Amman, JOR

**Keywords:** covid-19, systematic literature review, case review, covid-19 complications, infectious aortitis

## Abstract

Coronavirus disease 2019 (COVID-19) has a myriad of different presentations and various complications. Aortitis is one of the less explored pathologies associated with COVID-19 infections. In this review, we searched PubMed/Medline, Web of Science, Google Scholar, and Scopus for case series and case reports involving adults patients who presented with aortitis and COVID-19. We found and reviewed four published case reports of aortitis in a setting of COVID-19 infection. The mean age of the four adult patients was 69 ± 1.732 years (range = 63-71 years), and all patients were males. Most of the patients (75%) did not have any preexisting comorbidities. All patients were treated conservatively and recovered with excellent outcomes.

## Introduction and background

A novel coronavirus, first reported in Wuhan, China, on December 31, 2019, emerged to become a global pandemic, as declared by the World Health Organization (WHO) in March 2020. According to the Centers for Disease Control and Prevention, the illness caused by the novel severe acute respiratory syndrome coronavirus 2 (SARS-CoV-2) can manifest in different organ systems, with a severity ranging from a mild to life-threatening illness. In 2020, coronavirus disease 2019 (COVID-19) was the third leading cause of death in the United States [1].

Aortitis is a pathologic term referring to all infectious and non-infectious conditions that lead to the inflammation of the aortic wall [2]. Patients often present with non-specific symptoms posing challenges to diagnosis [3]. Pyogenic aortitis caused by *Salmonella* and *Staphylococcus aureus* infections is the most common form of infectious aortitis, whereas non-infectious aortitis includes Takayasu arteritis and giant cell arteritis [2].

Infectious aortitis refers to any inflammatory process of the aortic wall involving an infectious agent [3]. Aortic intima is generally immune to infection; however, certain aortic disorders, such as atherosclerotic plaques, preexisting aneurysms, trauma, and conditions that impair immunity (e.g., diabetes, immunosuppression, and cancer), can weaken the aortic wall and predispose to aortitis [4]. Various etiologies dictate the variable presentations and outcomes of aortitis patients; thus, a thorough workup is required to help guide clinicians toward the optimal management regimen. Little is known about aortitis in COVID-19 patients; therefore, we reviewed all published cases of COVID-19 patients with a diagnosis of aortitis, along with relevant clinical presentations, management, and outcomes.

## Review

Methodology

We searched PubMed/Medline, Web of Science, Google Scholar, and Scopus until March 19, 2021, for case reports and case series using the following keywords: COVID-19, SARS-CoV-2, aortitis, and large vessel vasculitis. In the final analysis, we only included case reports published in the English language. Eligible case reports and case series were reviewed by two independent researchers, and any conflict was resolved by a third researcher. Our search identified 359 studies in total. After removing duplicates and selecting case reports or case series with individual patient-level data, we identified seven articles. Due to the young age of patients, we further excluded two articles. Another article was excluded due to the lack of viral and serologic evidence of COVID-19 infection. Finally, we included four articles (Table 1) [5-8]. Continuous variables were presented as means and standard deviations, and categorical variables as absolute values and percentages. Microsoft Excel was used for data extraction and to perform all descriptive analyses.

**Table 1:** Case reports included in this review of published case reports. CECT: contrast-enhanced computed tomography; COVID-19: coronavirus disease 2019; CT: computed tomography; DM: diabetes mellitus; HTN: hypertension; IgG: immunoglobulin G; NSAIDs: non-steroidal anti-inflammatory drugs; PCR: polymerase chain reaction

Author	Age	Sex	Medical history	COVID-19 diagnosis	Aortic segment involved	Mode of diagnosis of aortitis	Treatment
Dhakal et al. 2021 [5]	63	M	Parkinson’s disease, asthma, DM, HTN, non-ischemic cardiomyopathy	Positive PCR	Infrarenal aorta	CECT	Prednisone 60 mg
Shergill et al. 2020 [7]	71	M	None	Positive IgG	Subclavian arteries to the iliac bifurcation	CT	Prednisolone 40 mg
Oda et al. 2020 [6]	71	M	None	Positive PCR	Abdominal aorta to the bilateral common iliac arteries	CECT	NSAIDs
Zou and Vasta 2020 [8]	71	M	Cholecystectomy, rotator cuff repair	Positive IgG	Aortic arch extending all the way down the aorta	CT	Prednisolone 40 mg

Results

We identified four articles through our search involving a total of four COVID-19 patients. The mean age of the patients was 69 years (63-71 years, SD = 3.464). All patients were males (100%). Two of the reported cases were from the United Kingdom, one was from the United States, and one was from Japan. Race was not included in the analysis because it was not reported in all the case reports. We could not discern a distinct set of comorbidities that might predispose patients to aortitis.

Overall, two (50%) patients presented with an active COVID-19 infection detected on polymerase chain reaction with COVID-19 symptoms at the time of presentation, while the other two had serological evidence of past infection with COVID-19. Regarding presenting symptoms, out of the four patients, two (50%) presented with fever. Further, three (75%) patients reported generalized weakness, three (75%) reported pain (chest pain, back pain, and abdominal pain) (75%), and two (50%) presented with weight loss. Three (75%) patients manifested symptoms that triggered the search for an alternative diagnosis following the resolution of the respiratory symptoms. Physical examination was unremarkable in three (75%) patients and was only significant for abdominal discomfort on palpation in one (25%) patient.

Patients consistently presented with a markedly elevated C-reactive protein at the time of diagnosis with a mean of 142.75 ± 23.3 mg/L (range = 87-185 mg/L, SD = 46.5). The immunologic screen was negative except for elevated immunoglobulin G4 in two patients who presented with an acute COVID-19 infection. CT was used in all four patients to detect inflammation in large vessels, and fluorodeoxyglucose positron emission tomography/computed tomography was performed for one (25%) patient. All four patients failed to illicit an infectious or rheumatologic cause for their aortitis, although one of them tested positive for human leukocyte antigen-DR4.

Prednisolone (40 mg and 60 mg) was used in most patients 75% (n = 3), while one patient only received non-steroidal anti-inflammatory drugs (NSAIDs). Resolution of inflammation, as evident on the repeat CT scans, was reported in all four patients, with two patients showing complete resolution [5,6], and the other two patients showing partial resolution [7,8]. The patient who was only treated with NSAIDs had a longer course of illness (24 days post-admission) [6]. In one patient, the symptoms persisted for three months before the initiation of steroid therapy [8]. All patients had a favorable disease course and recovered without any adverse events.

Discussion

To our knowledge, this is the first systematic review of COVID-19 patients developing aortitis. Several viral infections have been linked to aortitis. Fukunaga et al. reported a case of aortitis as a rare complication secondary to hepatitis C virus-associated cryoglobulinemia [9]. Other viruses such as hepatitis B virus, cytomegalovirus, Epstein-Barr virus, parvovirus B19, and human immunodeficiency virus can also be associated with the development of large-vessel vasculitis [10,11]. It is not always clear whether viral processes, antiviral side effects, or secondary bacterial vasculitis caused by immune suppression are involved in the pathogenesis of aortitis [12].

The main findings of this review are that all four adult aortitis patients were males and older than 60 years. Only one of the patients had a current history of underlying cardiovascular comorbidities, namely, non-ischemic cardiomyopathy, hypertension, and diabetes. The time of presentation since the acute COVID-19 infection varied widely, with two patients presenting after two months of the acute infection while the other two presenting along with the onset of the acute infection.

All three patients who received prednisolone improved rapidly with recovery times of less than two weeks. One patient was treated with NSAIDs because of the fear of COVID-19 reactivation early in the pandemic when data about the disease were lacking. However, he took approximately a month to recover. This highlights the importance of high-dose steroids in the management of aortitis in the setting of COVID-19. Furthermore, this was supported by the duration of symptoms before treatment in the patient who presented after three months of symptom onset and then improved shortly after starting steroids.

We noticed that all patients who presented with the acute infection had their disease limited to shorter segments of the aorta on CT compared to patients with late presentation who had a more diffuse type of aortitis involving the entire aorta.

Although the mechanism of aortitis in COVID-19 is not yet fully clarified, a histological case series found that SARS-CoV-2 can directly infect endothelial cells, probably through endothelial angiotensin-converting enzyme 2 receptors, causing endothelial inflammation [13]. Alternatively, Zou et al. speculated that the cytokine storm that can be seen in some COVID-19-infected patients causes endothelial cell dysfunction and inflammation in the setting of elevated interleukin-6 which was observed in two of the cases we reviewed [8]. However, this does not explain the onset of aortitis symptoms appearing after the resolution of the initial viral infection in two of the patients included in this review. This leads us to propose that aortitis can be mediated by an immune response rather than as a direct consequence of the virus.

Limitations

COVID-19 is known to produce a hypercoagulable state [14]; however, cases of aortic thrombosis were excluded even if they were complicated with aortitis. This can underestimate the gravity of the condition in infected individuals. Because the published literature about aortitis in the setting of COVID-19 is limited, any inferences made from this review should be interpreted carefully.

Dexamethasone has been shown to be beneficial in COVID-19 infection management [15] and has been recommended by several guidance bodies. It is unknown whether the use of dexamethasone would affect the presentation of aortitis in COVID-19-infected patients.

## Conclusions

We propose screening patients with prolonged generalized symptoms, abdominal discomfort, unexplained persistent spiking fevers, or persistently elevated inflammatory markers for aortitis by abdominal imaging (CT scanning) because this condition is readily treatable in adults. It appears that high-dose steroids are effective in managing aortitis associated with COVID-19 infection. Further studies are needed to confirm this hypothesis; however, clinicians should be aware of this complication when managing COVID-19 patients.
